# Trisenox disrupts MDM2-DAXX-HAUSP complex and activates p53, cell cycle regulation and apoptosis in acute leukemia cells

**DOI:** 10.18632/oncotarget.26025

**Published:** 2018-09-04

**Authors:** Sanjay Kumar, Andrea Brown, Paul B. Tchounwou

**Affiliations:** ^1^ Cellomics and Toxicogenomics Research Laboratory, NIH/NIMHD-RCMI Center for Environmental Health, College of Science, Engineering and Technology, Jackson State University, Jackson, Mississippi, MS 39217, USA

**Keywords:** trisenox, APL, MDM2-DAXX-HAUSP complex, p53, apoptosis

## Abstract

Trisenox (TX) has been used in the treatment of both *de novo* and relapsed acute promyelocytic leukemia (APL) patients. Using *in vitro* APL cell lines model in this research, we report on a new target of TX action through disruption of MDM2-DAXX-HAUSP complex, degradation of MDM2, and activation of p53 expression. TX–induced stress signal was transmitted by protein kinase (ATM & ATR) and phosphorylation of its downstream targets CHK1, CHK2, ATM, and ATR, respectively at the Ser 345, Thr68, Ser1981 and Ser 428 residues involved in complex disruption and p53 up-regulation. TX-activated p53 led to cell cycle arrest and apoptosis in APL cells. Our results showed that TX inhibited cell proliferation, disrupted complex molecules expression and association in APL cells. Our functional studies indicated that TX-induced down-regulation of complex molecules expression was mostly neutralized in both p53 knockdown NB4 cells and nutilin-3 treated KG1a cells. Hence our findings provide a functional evidence of TX action on cell cycle regulation and apoptosis in APL cells. This novel target of TX activity may be useful for designing new APL drugs.

## INTRODUCTION

APL, a blood cancer characterized by a translocation mutation between chromosome 15 and chromosome 17, strikes about 1,500 patients in the United States annually [1, 2]. This mutation leads to the formation of two fusion genes (oncogenes), promyelocytic leukemia– retinoic acid receptor alpha (PML–RARα) and RARα–PML. PML–RARα fusion transcript is involved in pathogenesis of APL whereas RARα–PML fusion transcript, an important molecular marker for the diagnosis and monitoring of APL [2, 3]. Current treatment options for APL patients include: all - trans retinoic acid (ATRA), combination of ATRA and anthracyclines, ATRA and arsenic trioxide (TX) combination, and TX alone. ATRA acts through induction of differentiation of leukemic promyelocytes into mature granulocytes in APL patients [4–7]. It also degrades promyelocytic leukemia (PML)-retinoic acid receptor-α (PML-RARA) fusion protein and eradicates leukemia–initiating cells in mouse model of APL [8]. The advent of ATRA in APL pathophysiology has made revolutionary changes in APL patient’s treatment strategies and almost ended previous life-threatening chemotherapy treatment with 6-mercatopurine (6-MP), methyl-glyoxal guanyl hydrazine, daunorubicin (DNR), and cytarabine [9]. It has been reported to induce complete remission (CR) rates of 85% from investigations conducted by the Shanghai group [7], and of 72% from studies done by the North American Intergroup [4]. However, APL patients often relapse from ATRA treatment due to excessive clearance and lower blood plasma level in course of treatment. Also, ATRA is good mainly for low risk APL patients [9, 10]. It may cause life-threatening complication of ATRA syndrome [11]. Moreover, ATRA works only PML-RARAα containing APL patients, and not in RA-resistant promyelocytic leukemia zinc finger (PLZF)-RARα patients [8]. Combination of ATRA and anthracyclines have successfully improved the relapse rate and lowered the incidence of ATRA syndrome in APL patients [9, 11]. These combinations also enhance the anti-leukemic efficacy of APL patients by raising CR rates to more than 90%, leading to a virtual absence of resistance, and a survival rate of nearly 80% [12]. They are considered the standard induction treatment for newly diagnosed APL patients [13]. Although the ombination of ATRA and anthracyclines is used prominently for low risk APL patients in induction therapy, it not able to cure relapsed or refractory patients properly, and also it is not effective in the treatment of APL patients all age groups for longer survival [9, 12, 14]. ATRA and arsenic trioxide (ATO) combination is a targeted therapy without inclusion of chemotherapy. This combination acts through induction of maturation of promyelocytes, apoptosis, and eradication of APL-initiating cells through degradation of PML-RARAα [15–18]. In consolidation therapy APL patients undergoing ATRA-ATO combination treatment show improved CR rate between around 90-95% with a decrease in relapse rate [19]. ATRA-ATO combination has also emerged as a standard care for both low and intermediate risk APL patients, with possible superior outcomes. It is also best suited for older and cardiovascular disease-complicated APL patients [20]. ATRA-ATO combination is not recommended to high risk refractory APL patients. It also does not work in RA-resistant promyelocytic leukemia zinc finger (PLZF)-RARα patients and may causes some sort of cardiovascular problems [8, 9, 12, 14]. TX is the preferred drug of choice for relapsed or refractory APL patients of all age groups. It is used in both induction and consolidation therapy and also high-risk APL patients with combination of ATRA and idarubicin, providing a maximum CR (∼ 99 %) with longer survival rate [9, 12, 14, 21]. TX acts inside APL cells by various mechanisms, producing reactive oxygen species (ROS), oxidative stress, DNA damage, and p53 activation leading to cell cycle regulation/ arrest and apoptosis [9, 12, 14, 21–23]. However, few cases of TX drug resistance have been reported in some APL patients with different fusion gene/oncogene such as PLZF-RARα responsible for pathogenesis of APL [24, 25]. Hence, there exists a need to find new targets of TX action that would help in designing new drugs to cure APL patients quickly and overcome drug resistance. P53 induces cell cycle arrest and apoptosis in response of genotoxic and other stresses in several cancer cells [26, 27]. Its expression level is kept normal in cells by several ubiquitin ligases (E3), predominantly mouse double minute 2(Mdm2) and Mdm4/MDMX, through ubiquitination and proteasomal degradation. MDM2 is a negative regulator of p53 that interacts with DAXX and HAUSP to form tertiary complex (MDM2-DAXX-HAUSP) [28]. Tertiary complex reduces self-ubiquitination of MDM2, maintaining MDM2 ligase activity toward p53 inside the cell. During cellular stress, complex is disrupted, increasing MDM2 self-ubiquitination and degradation, which leads to accumulation of p53 [28–30]. Accumulating evidence suggests that TX inhibits proliferation through activation of p53, p21, cell cycle arrest, and apoptosis in fibroblast cells [23], human gastric cancer cells [31], human myeloma cells [32], HL-60 cells [21, 33], lymphoid malignant cells [34], and NB4 cells [35]. TX promotes accumulation of DAXX, which modulates transcription of death-related genes in apoptosis [36]. Berberine, doxorubicin, and VP-16 kill cancer cells through disruption of complex, self-ubiquitination and degradation of MDM2, which leads to accumulation of p53 [28, 37]. Promyelocytic leukemia (PML) gene co-localizes with p53 and is involved in pro-apoptotic events [38]. Promyelocytic leukemia zinc finger-retinoic acid receptor α (PLZF-RARα) promotes cell proliferation in APL patients by repression of p53 and p21 proteins expression [39]. PML-transformation related protein (Trp53) is required to control leukemia –initiating cells in mouse model of APL [8]. Pseudokinase Tribble 3 (TRIB3) promotes APL progression by inhibition of p53 mediated senescence. Arsenic trioxide interacts with TRIB3/ PML–RARα and eradicates APL [40]. Nutlin-3, an antagonist of MDM2 induces p53 dependent apoptosis in acute lymphoblastic leukemia cells [41, 42]. In the present research, we discovered a new target of action of TX through transmission of stress signal with protein kinases, and their downstream phosphorylation targets, disruption of MDM2-DAXX-HAUSP complex, and activation of p53 leading to cell cycle regulation and apoptosis in APL cell lines.

## RESULTS

### Trisenox inhibits APL cells proliferation

**Figure 1:**
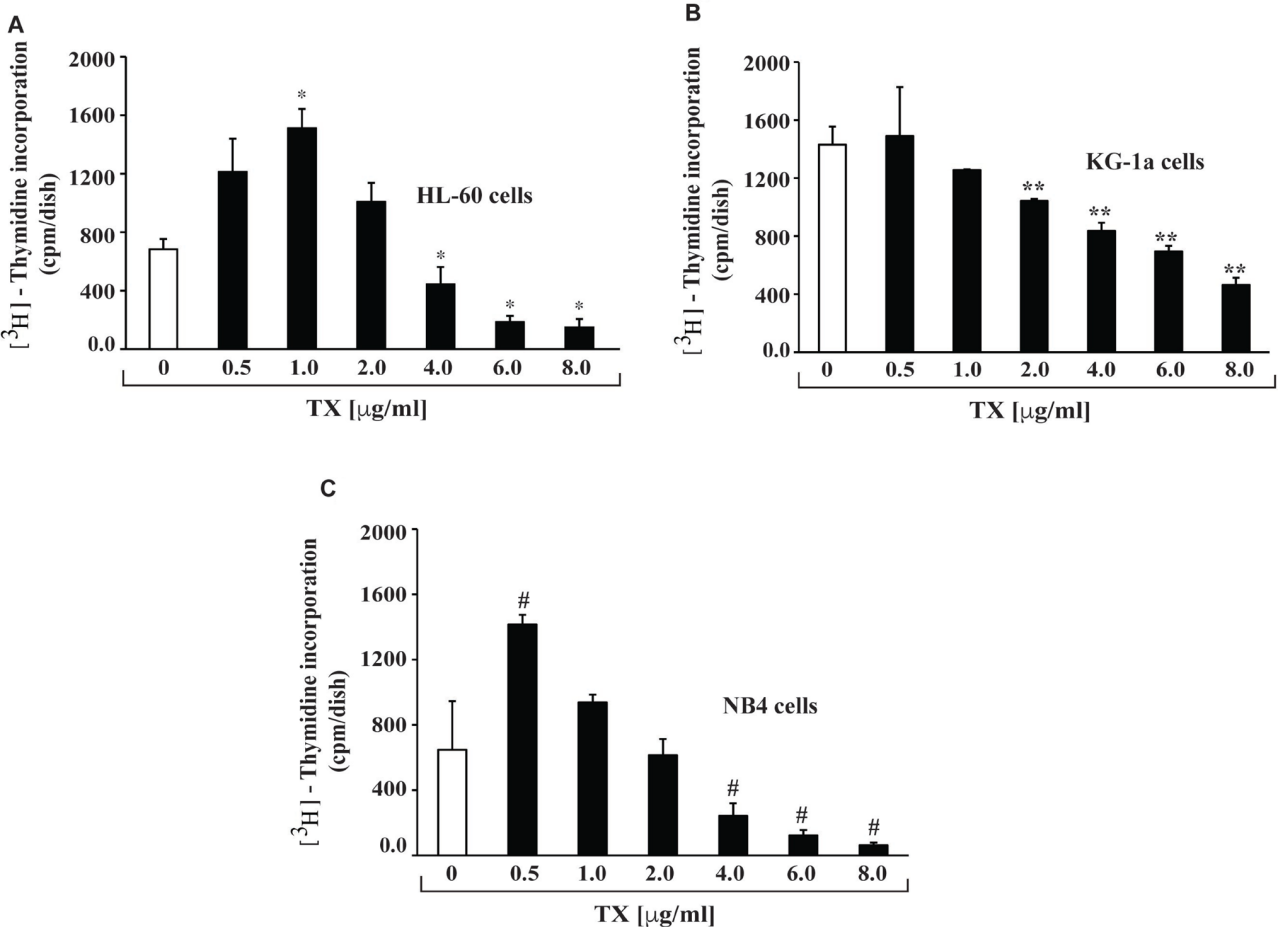
TX inhibits proliferation of APL cells APL cell lines were treated with different concentrations of TX for 24 hours and 21 hours with tritium labeled thymidine. After incubation, cells were harvested, counted, and ^3^H-methyl thymidine incorporation expressed as cpm/dish. Highly statistically significant decreases (*p* < 0.01) in cell proliferation were observed in HL-60 (**A**), KG-1a (**B**) and KG-1a (**C**).

To investigate the effect of TX on APL cells growth, we conducted cell proliferation assay using methyl thymidine incorporation protocol. Cells were treated to various concentrations (0, 0.5, 1,2, 4, 6 and 8 µg/ml) of TX for 24 hours and then further incubated with tritium labeled methyl thymidine for 21 hours. Study results indicated that TX stimulated cell proliferation up to one µg/ml and inhibited cells growth significantly (*p* < 0.01) at high doses (4, 6 and 8 µg/ml) of TX in a dose - dependent manner, in all three APL cell lines (Figure 1A–1C) tested.

### Trisenox activates p53 expression and cell cycle arrest

**Figure 2:**
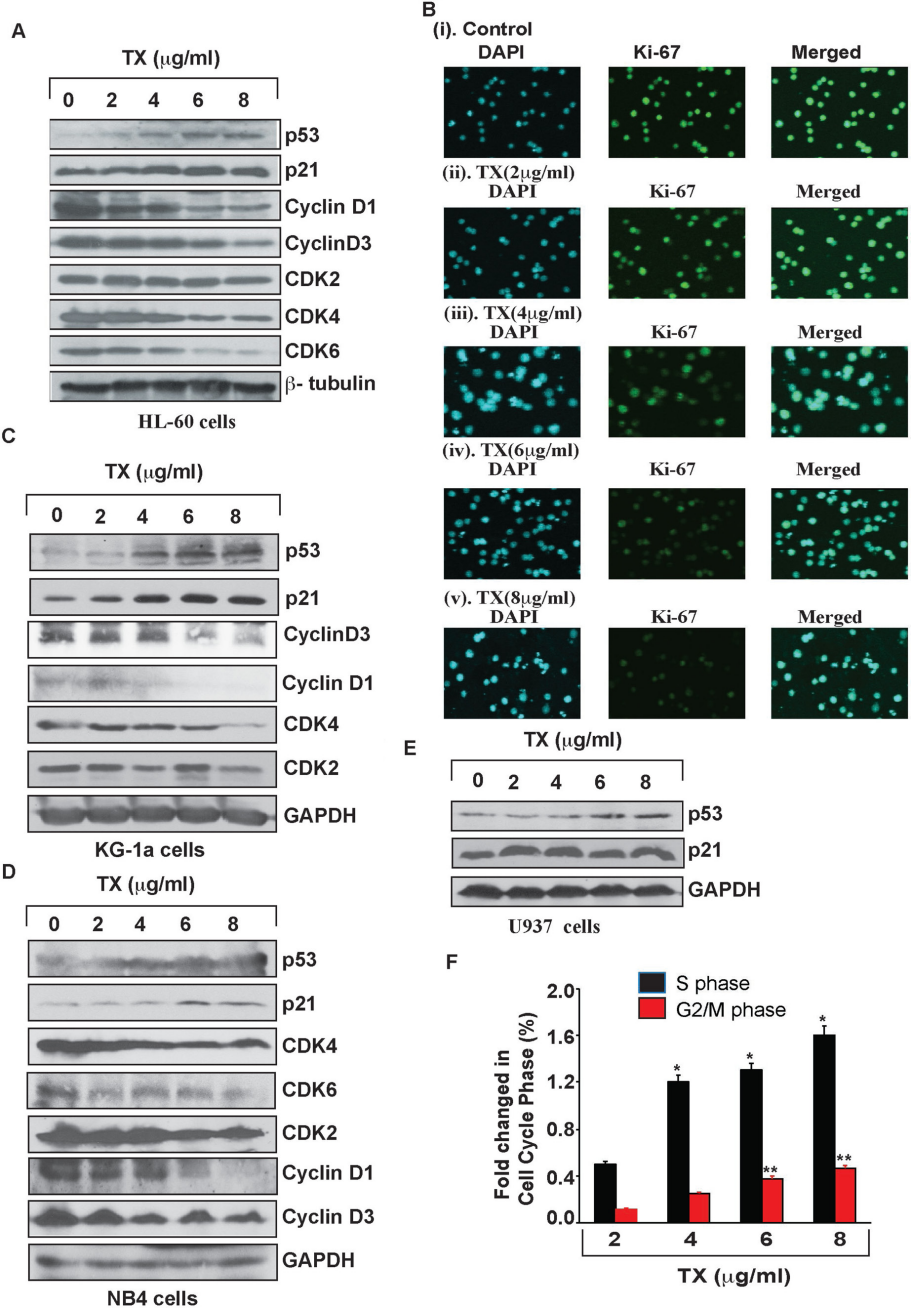
TX activates p53 leading to cell cycle arrest TX activated p53 and p21 through down regulation of expression of cyclins, cdks, and ki67 in HL-60 (**A**), KG-1a (**C**), NB4 (**D**), and U937 (**E**). TX induced cell cycle arrest at S and G2/M phase prominently (**F**) in APL cells.

To study TX –induced p53 accumulation and cell cycle arrest of APL cells, we treated Hl-60, KG1a, NB4 and U937 with various concentrations (2, 4, 6 and 8 µg/ml) of TX for 24 hours and analyzed cell cycle regulatory proteins expression by Western blotting. Interestingly, p53 and p21 expressions were stimulated significantly in TX treated APL cells compared to untreated cells (Figure 2A, 2C–2E). Whereas cyclins (cyclin D1&3) and cyclin dependent kinases (CDK2, 4 &6) expressions were down regulated concentration - dependently in APL cells treated with TX (Figure 2A, 2C–2D). Our immunocytochemistry experiment also showed a significant reduction in ki67 expression TX treated APL cells (Figure 2B (i-v)). Overall, TX induced accumulation of p53, p21 leading to cell cycle arrest, which was also confirmed by a significant decrease in the expression of ki67 and increase in the percentage of cell cycle arrest at S & G2 phase (Figure 2F).

### Trisenox induces intrinsic pathway of apoptosis

**Figure 3:**
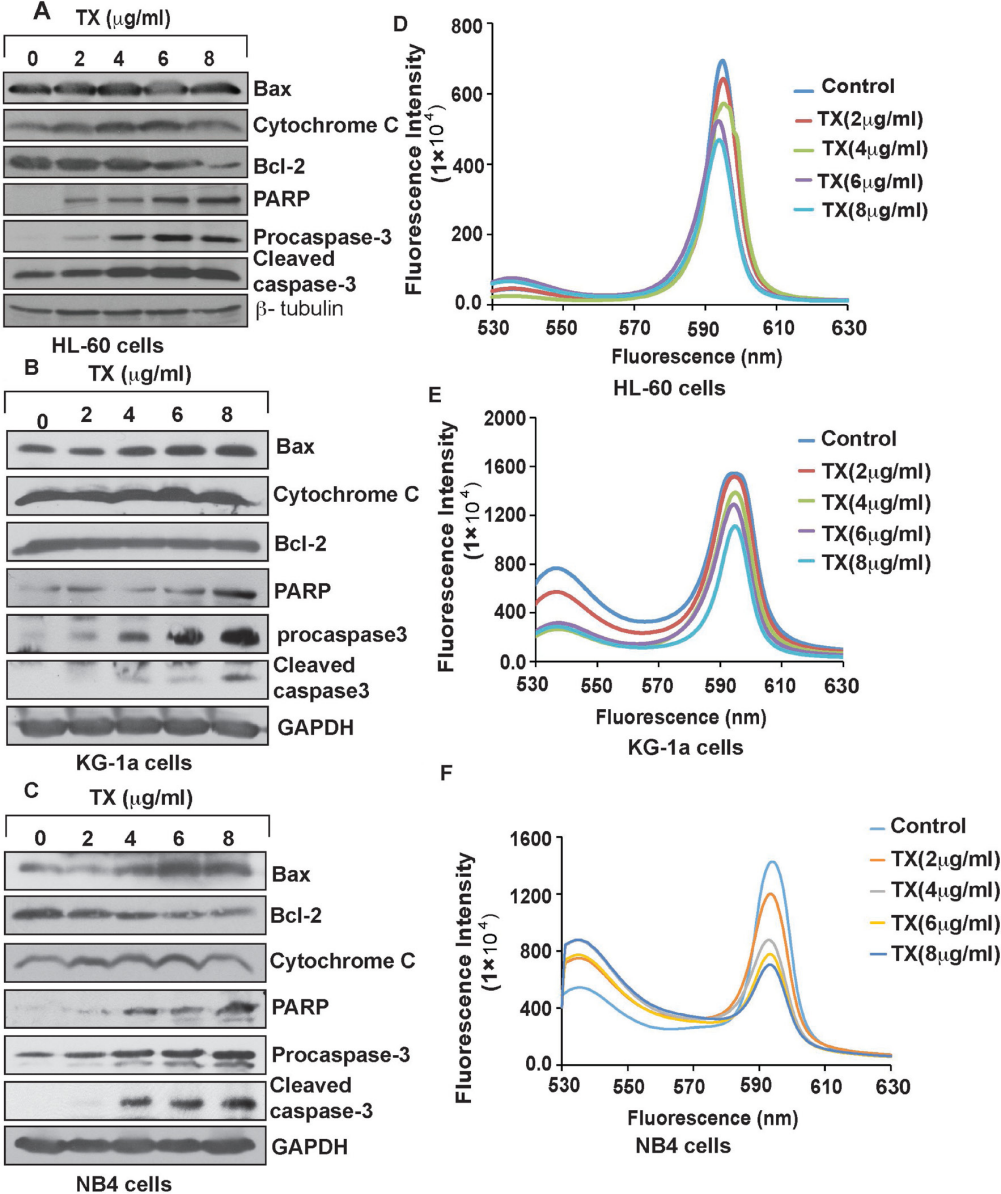
TX induces intrinsic pathway of apoptosis TX upregulated the expression levels of Bax, Cytochrome C, PARP, and caspase 3 through down regulation of Bcl-2 expression in HL-60 (**A**), KG-1a (**B**), and NB4 (**C**). Mitochondria were isolated from samples and mitochondrial membrane potentials [MMPs] were measured by spectrofluoremetry. TX was reduced the MMPs in HL-60 (**D**), KG-1a (**E**) and NB4 (**F**) cells.

We investigated TX-induced p53 mediated apoptosis in APL cells by Western blot analysis of the expression levels of proapoptotic proteins and Bcl-2 by Western blotting both in all samples. Our finding showed that p53 stimulated the expression levels of pro-apoptotic proteins and also down regulated the expression levels of anti-apoptotic proteins (Figure 3A–3C). Our experimental results showed that TX –induced p53 significantly decreased mitochondrial membrane potential (Figure 3D–3F) in APL cells.

### Trisenox disrupts MDM2- DAXX-HAUSP complex

**Figure 4:**
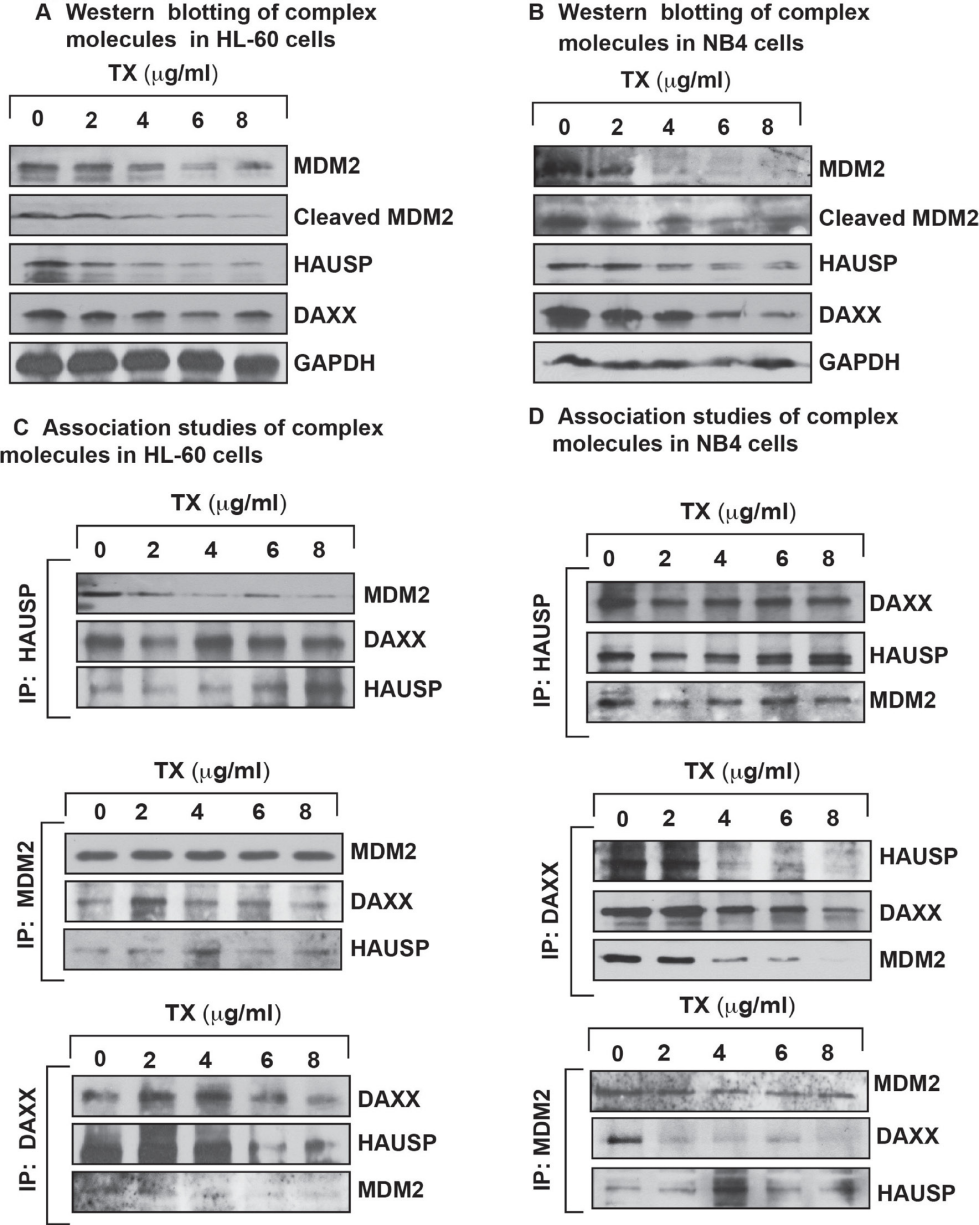
TX disrupts complex molecules expression and association TX effect on both expression and association of complex molecules in APL cell lines was analyzed by Western blotting and immunoprecipitation (IP) method. TX disrupted/changed the expression level of complex molecules in a concentration dependent manner and changed association level of complex molecules in HL-60 (**A** and **C**) and NB4 (**B** and **D**) cells.

To investigate TX effect on complex disruption with interaction of DAXX and degradation of MDM2 in APL cells, we treated HL-60 and NB4 cells with different concentrations of TX and analyzed complex disruption and interaction through immunoprecipitation (IP) and Western blotting. Our experimental results revealed that TX down regulated the expression level of complex molecules in both cells (Figure 4A, 4B). Also, IP results indicated that all complex molecules were associated with each other in APL cells (Figure 4C, 4D).

### Functional studies of Trisenox in complex disruption

**Figure 5:**
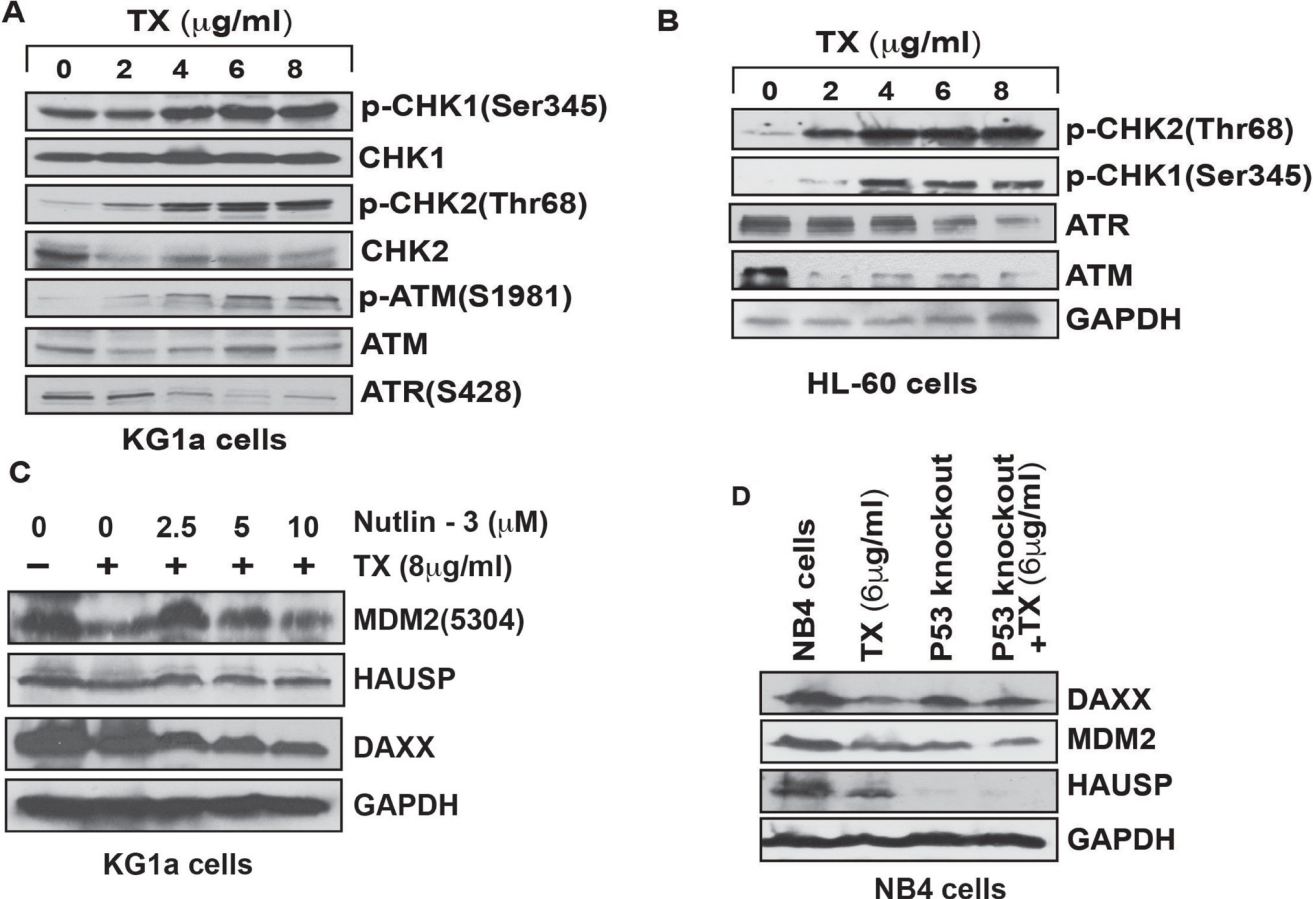
Functional mechanism of TX in complex disruption TX induced the phosphorylation of CHK1, CHK2, ATM, and ATR at different residues and changed the expression levels of ATM and ATR in both KG1a cells and HL-60 cells (**A**, **B**). Nutilin-3, an antagonist of MDM2 neutralized TX repression of MDM2 without effecting DAXX and HAUSP expression (**C**). TX treatment did not significantly reduce the expression of complex molecules in p53-knockout NB4 cells (**D**).

TX–induced stress signal involved in complex disruption is transmitted though stimulation of phosphorylation of CHK1 at Ser 345 residue, CHK2 at Thr 68 residue ATM at Ser 1981 residue and ATR at Ser428 residue in both KG1a and HL-60 cells (Figure 5A, 5B). We evaluated the functional significance of p53 in MDM2-DAXX-HAUSP complex molecules expression by Western blotting. TX reduced the expression level of complex molecules in normal NB4 cells, but in p53 knock-down cells, the expression was not significantly reduced (Figure 5D). We also assessed the role of nutlin-3 in overcoming TX effect in complex disruption. We found that nutrilin-3 neutralized TX-induced down-regulation of complex molecules in KG1a cells (Figure 5C).

## DISCUSSION

Generally, TX enters into APL cells through diffusion and acts by various mechanisms in different pathways [21, 23, 43, 44]. Previous studies have reported that it induces differentiation at lower doses, but at higher doses, produces cell cycle arrest and apoptosis in APL cells [44, 45]. Earlier studies suggested that it interacts with p53 and co-localizes together into PML nuclear body (PML-NB) leading to apoptosis [38, 46, 47]. In most of human cancers, p53 is mutated or remains functionally inactive by MDM2 and MDMX through E3 ligase activity. Reactivation of p53 could be an attractive and effective cancer therapy [48]. TX-induced p53 mediated cell cycle arrest and apoptosis in APL cells/ tissues mostly remains unknown. We used four APL cell lines to investigate TX-induced molecular mechanisms of action involving p53 activation, MDM2-DAXX-HAUSP complex disruption, cell cycle regulation, and apoptosis. Some of our gene expression research data are not consistent across all cell lines. This may be due to the source origin, genotype, and/or culture conditions of each specific APL cell line. Our findings indicate that both HL-60 and NB4 cells underwent proliferation at lower doses of TX, but this proliferation was significantly inhibited at higher doses of TX (Figure 1A–1C). Existing evidence suggests that TX induced cell cycle arrest at G1 or G2/M phase in several cell types [22, 23, 31, 49] by down regulation of cyclins and cdks expression [32]. Our finding provides additional evidence that TX activated the expression of p53 and p21 involved in cell cycle arrest prominently at S and G2/M phase, through down regulation of expression of ki67, cyclins and cdks in APL cell lines (Figure 2A–2F). Accumulating evidence also suggests that TX-induced p53 up-regulates the intrinsic pathway of apoptosis in several cell types [21, 23, 31]. Our data show that TX induced p53 was linked to the intrinsic pathway of apoptosis (Figure 3A–3F).

Several research groups have reported that p53 is accumulated in response of cellular stress and DNA damage due to DAXX interaction, MDM2-DAXX-HAUSP complex disruption and MDM2 degradation inside the stressed cells [28, 29, 30, 37]. Our new findings provide evidence that TX disrupted complex through reduced expression of complex molecules, associations, and degraded MDM2 in both APL cells (Figure 4A–4D). Accumulating evidence also suggests that p53 expression level and activity are controlled by MDM2 E3 ubiquitin-ligase through recruitment of E2 ubiquitin-conjugating enzymes to transfer ubiquitins onto p53 [50, 51]. Our findings show that TX-induced stress signal is transmitted through modulation of significantly phosphorylation of CHK1 at Ser345 residue, CHK2 at residue Thr68, and ATM at residue Ser 1981 as well as changed expression of ATM & ATR in both KG1a and HL-60 cells (Figure 5A, 5B). This resulted in complex disruption, MDM2 degradation, and accumulation of p53 in both APL cells. (Figure 2A, 2C–2E). Our functional studies data show that TX treatment did not significantly reduce the expression of complex molecules in p53 knock-down NB4 cells (Figure 5D). We also found that nutlin-3 overcame/neutralized TX–induced down-regulation of complex molecules in kG1a cells (Figure 5C).

**Figure 6:**
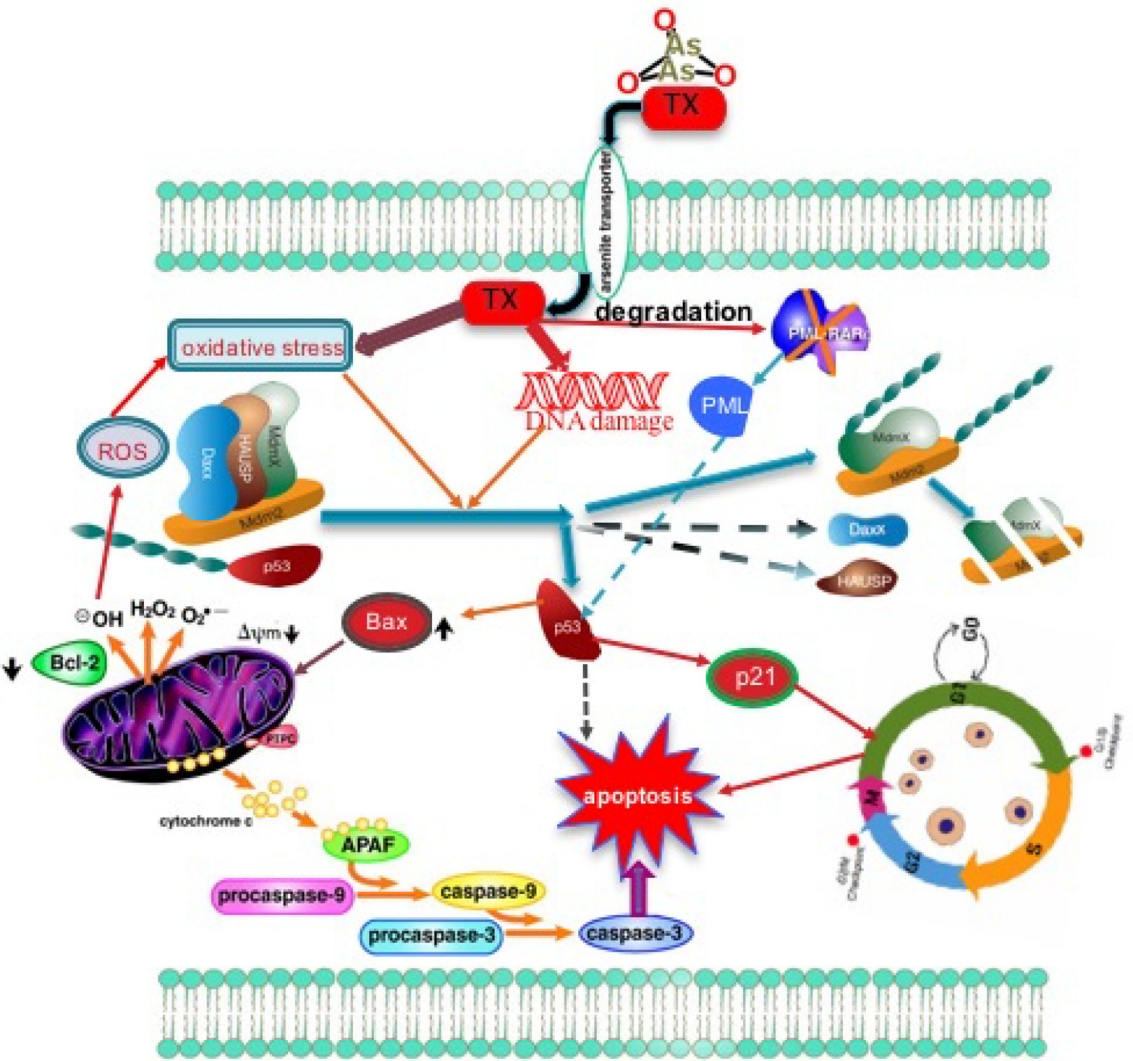
Summary of TX new target of action in APL cells TX enters in APL cells, and induces oxidative stress and DNA damage, leading to a reduced expression of complex molecules, MDM2 degradation through stress signal coordinated by protein kinases (ATM & ATR) and their downstream targets CHK1 and CHK2 residues, and subsequent activation of p53. TX-activated p53 induces cell cycle arrest in APL cells and forces them to undergo apoptosis.

In summary, we present here comprehensive molecular mechanisms of TX induced p53 activation via transmission of stress signaling by protein kinases [ATM, ATR] and their downstream targets, CHK1 and CHK2 residues phosphorylation leading to disruption of complex in APL cells (Figure 6). By elucidating the p53 role in TX-induced cell cycle arrest and apoptosis in APL cells, we have provided new insights into the molecular targets.

## MATERIALS AND METHODS

### Cell line and culture

HL-60, NB4, KG-1a and U937 cells, were used in this study. The cells were purchased from the American Type Culture Collection–ATCC (Manassas, VA), and maintained according to standard procedures. These four cell lines were chosen based on ATCC fact sheets, and on the fact that many previously published articles have used them as test models for APL research.

### Reagents

Trisenox (arsenic trioxide) was purchased from Fisher scientific (Waltham, MA) and thymidine (methyl-^3^H) was obtained from MP Biomedical (Santa Ana, CA). Nutlin – 3, Mitochondrial isolation kit, caspase assay kit, and protease inhibitor were obtained from Sigma-Aldrich (St. Louis, MO). anti-DAXX, anti-HAUSP, Anti-cytochrome C, anti-Bax and anti-Bcl2 were purchased by Cell Signaling Technology (Danvers, MA). Anti-MDM2 was purchased from Santa Cruz Biotechnology Inc.(Santa Cruz, CA). Hoechst 33342, Alexa fluor 568 and Alexa fluor 568 were purchased from Life Technologies (Grand Island, NY).

### Cell proliferation assay

APL cells were treated with different doses of TX in triplicate and labeled with thymidine methyl- ^3^H. Cells were harvested and the radioactivity measurement was performed using a liquid scintillation analyzer (Tri-carb 2700TR, Perkin Elmer) as previously described [2].

### Immunoprecipitation and Western blotting

After treatment with different doses of TX, APL cells were collected and protein lysates were prepared in RIPA buffer by sonication and centrifugation. We used 500ug protein lysate of APL cells for each sample. Western blotting and immunoprecipitation (IP) were performed as previously described [28].

### Measurement of mitochondrial membrane potential (Δѱm)

Mitochondria were isolated from all samples using mitochondria isolation kit (Sigma, St. Louis, MO, USA). Isolated mitochondria were incubated with 2 µl JC1 stain (from stock 1mg/ml) and 950 µl JC1 assay buffer for 10 min in dark at 25° C. The fluorescence of each sample was recorded using a Perkin Elmer LS50B spectrofluorometric (excitation 490 nm, slit, 5 nm; emission 590 nm, slit, 7.2 nm) [52].

### Cell cycle analysis

APL cells (1 × 10^7^ cells/ml) were treated with different doses (0, 2, 4, 6 and 8 µg/ml) of TX for 24 hours. After incubation, cells were harvested by centrifugation and the cell pellets were fixed in 500 µl ice cold ethanol for 15 minutes. Fixed cell pellets were mixed with 150 µl propidium iodide solution and incubated at 37° C for 40 minutes. After incubation, cell cycle analysis was done by Cellometer (Nexcelom Bioscience LLC, MA, USA) as described previously [53].

### Immunocytochemistry and confocal microscopy imaging

APL cells (1 × 10^5^) were cultured in presence or absence of TX and attached on poly-L-lysine coated slides. Immunocytochemistry of attached cells were performed using Ki67 antibody (dilution, 1:100) (cat# 33-4711) [54] or p53 antibody (cat # 9282) and PML-RARα (cat# ab43152) from Life Technology, Cell Signaling or Abcam company and imaged by confocal microscopy (Olympus Company, Center valley, PA) as described earlier [2].

### Knockdown of p53 in NB4 cells

We made p53 knockdown NB4 cells using lentivirus shRNA (Dharmacon Inc; Lafayette, CO) method as described earlier [55]. In brief, we seeded 10,000 NB4 cells in 25 µl of transduction medium (RPMI 1640) without serum in each well with polybrene (8 µg/ml). Then, we added 40 MOI SMART choice lentiviral p53 shRNA particles (10^5^ TU/ml) to each well and incubated for 20 hours at 37° C. After incubation, we added 75 ml of 20% serum containing culture medium in each well and further cultured for 2 days at 37° C. We performed microscopic examination and cell viability test and further incubated with puromycin (8 µg/ml) for a week. Puromycin-selected NB4 cells were further checked through Western blotting and fluorescence imagining. We used for our experiment more than 90% p53 knock-down NB4 cells.

### Statistical analysis

Experiments were performed in triplicates. Data were presented as means ± SDs and one-way ANOVA or student paired *t*-test using SAS Software available in the Biostatistics Core Laboratory at Jackson State University. *P*-values less than 0.05 were considered statistically significant.

## Abbreviations

APL: Acute promyelocytic leukemia
TX: Trisenox
P53: Tumor suppressor protein
DAXX: Death-associated protein 6
HAUSP: Herpesvirus-associated ubiquitin-specific protease
MDM2: Mouse double minute 2 homolog
JC1: 5,5′,6,6′-tetrachloro-1,1′,3,3′-tetraethylbenzimidazolylcarbocyanine iodide
DAPI: 4’,6-diamidino-2-phenylindole
PARP: poly ADP ribose polymerase
GAPDH: Glyceraldehyde 3-phosphate dehydrogenase
ATM: Ataxia-telangiectasia mutated
ATR: Ataxia telangiectasia and Rad3 related
CHK1: Checkpoint kinase 1
CHK2: Checkpoint kinase 2
PML-RARα: Promyelocytic leukemia- retinoic acid receptor alpha
ATRA: All trans retinoic acid
